# Maternal–Fetal Infections (Cytomegalovirus, *Toxoplasma*, Syphilis): Short-Term and Long-Term Neurodevelopmental Outcomes in Children Infected and Uninfected at Birth

**DOI:** 10.3390/pathogens11111278

**Published:** 2022-10-31

**Authors:** Cinzia Auriti, Silvia Bucci, Domenico Umberto De Rose, Luana Coltella, Alessandra Santisi, Ludovica Martini, Chiara Maddaloni, Iliana Bersani, Simona Lozzi, Francesca Campi, Concettina Pacifico, Martina Balestri, Daniela Longo, Teresa Grimaldi

**Affiliations:** 1 Medical and Surgical Department of Fetus-Newborn-Infant, “Bambino Gesù” Children’s Hospital IRCCS, 00165 Rome, Italy; 2 Department of Neurosciences, “Bambino Gesù” Children’s Hospital IRCCS, 00165 Rome, Italy; 3 Department of Microbiology and Virology, “Bambino Gesù” Children’s Hospital IRCCS, 00165 Rome, Italy; 4 Audiology and Otosurgery Unit, “Bambino Gesù” Children’s Hospital IRCCS, 00165 Rome, Italy; 5 Department of Imaging, “Bambino Gesù” Children’s Hospital IRCCS, 00165 Rome, Italy

**Keywords:** pregnancy, maternal infections, neonates, neurodevelopmental outcomes, CMV, *Toxoplasma gondii*, toxoplasmosis, *Treponema pallidum*

## Abstract

(1) Background: Infections in pregnancy can lead to miscarriage, premature birth, infections in newborns, and developmental disabilities in babies. Infected infants, symptomatic at birth, can have long-term sequelae, and asymptomatic babies are also at increased risk of developing long-term sensorineural outcomes. Targeted therapy of the pregnant mother can reduce fetal and neonatal harm. (2) Aim of the study: To explore the association between symptoms and time of onset of long-term sequelae in infected children born from mothers who contracted an infection during pregnancy, by a long-term multidisciplinary follow-up. (3) Methods: For up to 2–4 years, we evaluated cognitive, motor, audiological, visual, and language outcomes in infants with symptomatic and asymptomatic congenital infections and in uninfected infants. (4) Results: 186 infants born from women who acquired Cytomegalovirus infection (*n* = 103), *Toxoplasma* infection (*n* = 50), and Syphilis (*n* = 33) during pregnancy were observed. Among them, 119 infants acquired the infection in utero. Infected infants, symptomatic at birth, obtained lower scores on the Cognitive and Motor Scale on Bayley-III compared to asymptomatic and uninfected infants (*p* = 0.026; *p* = 0.049). Many severe or moderate sequelae rose up within the first year of life. At 24 months, we observed sequelae in 24.6% (14/57) of infected children classified as asymptomatic at birth, compared to 68.6% (24/35) of symptomatic ones (χ^2^ = 15.56; *p* < 0.001); (5) Conclusions: Infected babies symptomatic at birth have a worse prognosis than asymptomatic ones. Long-term sequelae may occur in infected children asymptomatic at birth after the first year of life. Multidisciplinary follow-up until 4–6 years of age should be performed in all infected children, regardless of the presence of symptoms at birth.

## 1. Introduction

Some maternal infections contracted before or during pregnancy can be transmitted to the fetus during gestation (congenital infection), during labor and childbirth (perinatal infection), and through breastfeeding (postnatal infection). The microorganisms most frequently responsible for congenital infections are Cytomegalovirus (CMV), *Toxoplasma gondii*, *Treponema pallidum*, Hepatitis B and C viruses, Human Immunodeficiency Virus, Parvovirus B19, and non-polio Enteroviruses [1,2]. Infections in pregnancy can damage the fetus (spontaneous abortion, fetal death, intrauterine growth retardation) or the newborn (congenital anomalies, organ diseases with sequelae of different severity) [3,4,5,6,7]. Some risk factors specifically influence the incidence of transmission to the fetus concerning the germ: the timing of the infection in pregnancy; the order of the infection; primary, or reinfection, or chronic; the duration of membrane rupture; type of delivery; socio-economic conditions; and breastfeeding [8,9,10]. To date, the maternal–fetal transmission of many microorganisms is preventable thanks to the use of specific drugs, vaccines, or passive immunization. Early therapy in pregnancy, when applicable, and post-natal care may improve the prognosis of infected neonates and prevent long-term sequelae [11]. When the mother is untreated, or the therapy is not applicable or not effective, infants with overt symptoms at birth have a worse prognosis than asymptomatic infants [2,12]. However asymptomatic neonates are also at an increased risk of delayed onset of mental and sensorineural sequelae. Most ocular and auditory damage can arise progressively over time [13]. These sequelae are often unpredictable without a long-term follow-up. Our study aimed to explore the association between symptoms of infection at birth and the occurrence and timing of long-term sequelae. We also evaluated the type of short- and long-term sequelae in our patients.

## 2. Materials and Methods

At the “Bambino Gesù” Children’s Hospital in Rome, there is an active multidisciplinary follow-up service and a specific outpatient clinic for newborns and children born to mothers who have contracted infections transmissible to the fetus during pregnancy. Infants with and without congenital infection from Cytomegalovirus, *Toxoplasma gondii*, and *Treponema pallidum* are prospectively monitored in a multidisciplinary follow-up of at least 24 months. The service aims to diagnose sensorineural deficit and development delay early and activate the rehabilitation measures quickly.

**Figure 1:**
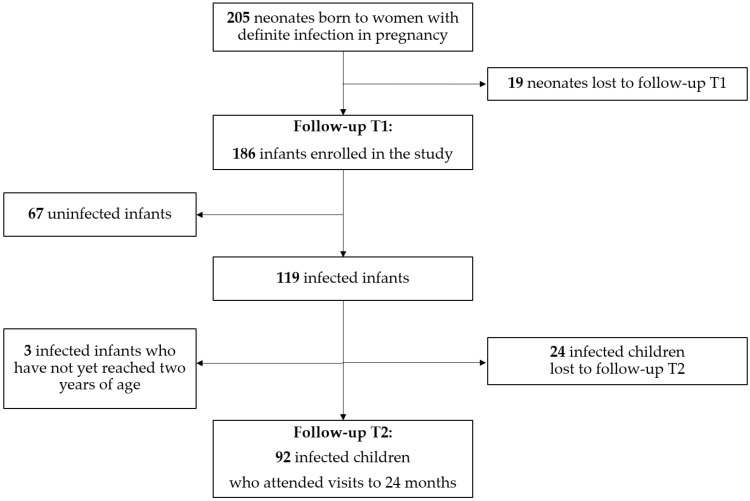
Flowchart of the study.

From December 2011 to November 2020, we longitudinally observed all consecutive infants born to women infected with Cytomegalovirus, *Toxoplasma gondii*, and Syphilis that had been referred to our clinic from other hospitals both located in the Lazio Region and other Italian Regions. Patients’ data were retrospectively obtained from medical records to assess short-term outcomes (at 12 months of life) and long-term outcomes (at 2–4 years of life) (Figure 1).

We collected information on infant condition at birth: gender, Apgar score at 5 min, birth weight, gestational age (GA), mode of delivery, ultrasonography, brain magnetic resonance imaging (MRI), hearing, and ophthalmologic examinations. Maternal infection-related variables were the time of the infection onset in pregnancy, laboratory values, and therapies, when available, as our institution does not have a delivery room and the women are referred to us by external service centers after giving birth. The retrospective study was reported to the Hospital Ethics Committee, which approved it.

### 2.1. Definitions

Infants were classified as symptomatic or asymptomatic if they had symptoms or not at birth for congenital CMV and Toxoplasmosis and high probable, possible, less likely, and unlikely congenital Syphilis. Table 1 shows the definitions of those congenital infections.

Clinically, the infants enrolled in the study were defined as small for gestational age (SGA) if their birthweight was less than the 10th percentile for gestational age. Head circumference was considered small for gestational age if its measurement at birth was less than the 10th percentile for gestational age. Infants were considered having a microcephaly if their head circumference was lower than the 3rd percentile.

Congenital CMV infection (cCMV) was defined according to the results of the consensus recommendations for prevention, diagnosis, and therapy by Rawlison et al. [14]. Asymptomatic infections were those with the identification of viral DNA in saliva, urine, or blood ideally during the first 3 weeks of postnatal life of neonates, without overt clinical symptoms of the infection at birth and normal hearing. Symptomatic infections were considered those with the presence of clinical symptoms for which the child was hospitalized or specific abnormalities of the brain US or of the brain magnetic resonance imaging (MRI) (ventriculomegaly larger than 10 mm, intracerebral calcifications, periventricular cysts, periventricular echogenicity, cerebellar or cortical malformations); abnormalities of the blood count (thrombocytopenia) which did not, however, lead to the need to hospitalize the child; and/or sensorineural hearing loss higher or equal to 21 decibels.

Symptomatic *Toxoplasma* congenital infections were diagnosed by clinical symptoms and serological exams during the hospitalization and, in asymptomatic neonates, by comparing and monitoring the serological maternal (specific IgG, IgG avidity, IgM) and neonatal status (specific IgG, IgM). Moreover, we performed a polymerase chain reaction for *Toxoplasma* DNA in serum at the first clinical evaluation. Overall, the neonatal infection was considered symptomatic in the presence of one or more of the following: intrauterine growth retardation, prematurity, petechiae, tachypnea, hepatomegaly, splenomegaly, thrombocytopenia, coagulopathy, seizures, microcephaly, hypotonia, chorioretinitis, abnormal skeletal radiography, hyperacusis, and retinitis. Associated main laboratory findings were thrombocytopenia and elevations in transaminases, in addition to specific laboratory tests for the disease. Microcephaly was considered when head circumference (HC) was below two standard deviations.

Congenital syphilis was diagnosed according to CDC criteria for confirmed, possible, less likely, and unlikely by clinical symptoms and, in asymptomatic neonates, by comparing the serological maternal status with that of the baby at birth. In neonates, we performed the polymerase chain reaction for treponemal DNA in serum and serological analysis: rapid plasma reagent, quantitative Treponema pallidum hemagglutination test (TPHA), specific IgG and IgM anti *Treponema pallidum*, and skeletal radiography, when appropriate. When the baby was hospitalized in the suspect of symptomatic syphilis and required therapy, we also performed a polymerase chain reaction for treponemal DNA in the cerebrospinal fluid.

All infected infants of this cohort were regularly evaluated to intercept the presence of a neurological disability early: the presence of cerebral palsy (CP) was defined according to Bax [15]. To evaluate neurodevelopmental outcomes, we used the Bayley Development Scale for Toddlers and Infants Third Edition (BSDI-III, 2006) [16]. This scale provides scores for three major development domains: cognition, language, and motor functions. Scores between 85 and 115 indicate normal development, while scores below 85 (−1 SD) indicate a developmental delay in the domain evaluated. The assessments were administered to patients by a developmental psychologist (S.B.) trained in BSID test procedures. The examiner was blinded to the neonatal course. The psychologist assessed neurodevelopmental outcomes at 12 and 24 months. Children with scores within the normal range in all three domains were considered normal; children with a score below 85 (−1 SD) in at least one of the three domains were deemed to be affected by a neurodevelopmental delay [17].

Repeated examinations of the fundus assessed visual function and retinal diseases. Normal vision was defined as the “absence of any detectable pathology of the visual system”; mild abnormal vision as “the presence of a mild impairment which allowed useful vision”; and severe visual impairment as “a child functionally blind or perceives light only” [18].

Hearing function was explored by automatic auditory brainstem response (AABR) and brainstem auditory evoked potentials (BAEPs). The global auditory function was defined as normal in the “absence of any detectable pathology”, as mild if requiring hearing aids or as severe if functionally deaf (uncorrected even with aids) [19].

### 2.2. Statistical Analysis

Data are shown as numbers and percentages for categorical variables, whereas continuous variables are expressed as mean ± standard deviation (SD) if they were normally distributed or as median and interquartile range if normality could not be accepted.

Clinical characteristics of the infants’ diagnosis at admission and the neurodevelopmental assessment in children with symptomatic, asymptomatic infected, and uninfected infants were compared using the Chi-squared test for categorical variables, whereas ANOVA was performed for continuous variables. A *p*-value < 0.05 was considered statistically significant. Statistical analysis was performed using software programs Microsoft Excel (2016 for Windows) and SPSS (version 20 for Windows).

## 3. Results

During the study period, we observed 205 infants born to women with definite infection during pregnancy. Of those babies, 19 were lost to follow-up before 12 months of age. Therefore, we considered for inclusion in this study 186 infants. Their mothers contracted Cytomegalovirus infection (*n* = 103/186; 55.4%), or *Toxoplasma gondii* infection (n = 50/186; 26.9%), or Syphilis (*n* = 33/186; 17.7%) during pregnancy. We did not observe infants from twin pregnancies. Among those 186 babies, 119 (64%) were affected by a confirmed maternal–fetal infection and the others resulted as uninfected. Among infected babies, 92/119 (77.3%) attended visits for 2–4 years.

The flowchart of the study is shown in Figure 1, while the clinical characteristics of all 186 babies enrolled in the study are summarized in Table 2.

Only 37/119 (31.1%) infected neonates were symptomatic at birth. Considering brain ultrasound (US) at birth, in 23/37 (62%) symptomatic neonates the brain US showed abnormal imaging findings related to the intrauterine infection, intracranial calcification included. Among asymptomatic neonates, 19/82 (23%) also showed brain US variations that were not considered by the neuroradiologist as specific or attributable and suggestive of fetal brain infectious involvement. In 4/67 uninfected neonates (6.0%), US showed mild cerebral ventriculomegaly lower than 10 mm, stable and non-evolutionary, cystic formation of the choroid plexus of the left ventricle (2 mm), and hyper-echogenicity of the periventricular deep white matter.

Among 119 infected babies, comparing the group of 37 symptomatic infants with that of 82 asymptomatic and with the 67 uninfected infants group, symptomatic infected babies have significantly lower gestational age, birth weight, smaller head circumference, and lower Apgar score at birth than the other groups of patients (respectively, F = 7.041, *p* = 0.001; F = 8.605, *p* < 0.001; F = 13.029, *p* < 0.001; F = 4.066, *p* = 0.019). No clinically significant differences were found between uninfected and asymptomatic infected babies at birth (Table 2).

In all, 17 neonates (9.1%) were born preterm; 10 (58.8%), 5 (29.4%), and 2 (11.8%) of them were among symptomatic infected babies, asymptomatic infected, and uninfected, respectively. The gender of newborns and the mode of delivery did not differ between groups.

Clinical characteristics of children did not differ concerning the type of maternal infection, except for CMV-infected infants, who presented lower birth weight and lower head circumference compared to other infants born to mothers with toxoplasma or syphilis infection (F = 3.578; *p* = 0.030).

A total of 58 infants (48.7%) received a specific pharmacological treatment: 31 (36.9%) neonates with CMV infection received intravenous ganciclovir and/or oral valganciclovir, 15 (83.3%) neonates with Toxoplasmosis received oral pyrimethamine and sulfadiazine and 12 (70.6%) received aqueous penicillin G intravenous treatment for congenital Syphilis.

### Outcomes in Children with Congenital Infection

**Figure 2:**
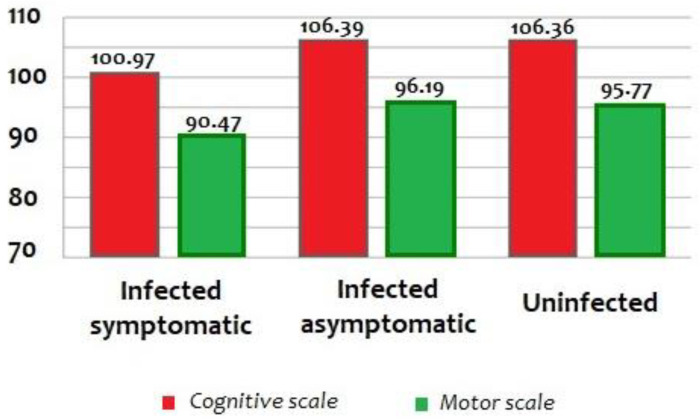
Cognitive and motor outcomes on Bayley Scales III in 186 children with symptomatic (*n* = 37), asymptomatic (*n* = 82), and uninfected *(n* = 67) at 12 months of life. *Bayley-III = Bayley Scales of Infant and Toddler Development, Third Edition*.

Overall, infected infants symptomatic at birth obtained lower scores on Bayley-III Cognitive and Motor Scales, than asymptomatic infected and uninfected infants (F = 3.73, *p* = 0.026; F = 3.07, *p* = 0.049), without any major differences related to the etiologic agent of the infection (Figure 2).

Concerning sequelae, at 12 months of life 90/119 (75.6%) infected children, symptomatic or asymptomatic at birth, resulted free from any sequela at the multidisciplinary clinical and instrumental evaluation, while the other 29 children (24.3%) showed one or more sequelae (Table 3).

The short-term outcome at Time-point 1 (T1, 1 year of life) in the cohort of 119 infected children, divided by the major development domains, visual and auditory function, is summarized in Table 4.

Overall, 4/119 (3.4%) children showed cognitive delay (Cognitive Scale < 85 on Bayley III and pathological neurological examination) already diagnosable before 12 months of life; 3 of them were born from mothers with Cytomegalovirus infection contracted early in pregnancy.

None of the children observed had cerebral palsy, but 17/119 (14.3%) had a motor delay (Motor Scale < 85). Again, most of those children 11/17 (64.7%) were born from pregnancies complicated by maternal Cytomegalovirus infection contracted early in pregnancy.

Additionally, 13 of 119 children (10.9%) had sensorineural hearing loss (SNHL), which was bilateral severe in only one case. There were no children with delayed-onset hearing loss, but 4 children showed a progressive SNHL, and 1/119 (0.8%) children had severe visual impairment.

Furthermore, 92 infected children were regularly observed up to 2–4 years of life: 4/27 (14.8%) of patients lost to follow-up had mild or mild unilateral hearing loss. After 2 years of life, 54/92 (58.7%) of infected children were free of sequelae during the follow-up. Their long-term outcomes at time point 2 (T2) are summarized in Table 5 and Table 6.

The most relevant aspects are the increase in cases of cognitive delay (3 new cases compared to the year of life) and above all the appearance of a considerable number of cases of delay in the language area (26/92, 28.3%) prevalent in those babies born to mothers with infection by Cytomegalovirus.

## 4. Discussion

In this study, we assessed the incidence and type of neurodevelopmental and sensorineural adverse outcomes of infants born to mothers infected in pregnancy by Cytomegalovirus, *Toxoplasma gondii*, and Syphilis. In total, 119/186 (64.0%) babies were infected at birth, with symptoms or not, globally considering them, beyond the specific pathogen, and separately. It is well known that up to two-thirds of cCMV-infected infants symptomatic at birth had long-term sequelae, in agreement with the literature reports (40–60%) [20,21], whereas it is interesting that up to one-fourth (24.6%) of infected infants without symptoms at birth developed them later in our cohort. This is a very important finding, as the need for therapeutic treatment of asymptomatic infants is an open question. Concerning cCMV infection, at present there is no evidence of the benefits of antiviral therapy in asymptomatic infants [22], and oral valganciclovir is administered to newborns with congenital infection and severe symptomatic disease at birth, such as microcephaly, intracranial calcifications, cerebrospinal fluid abnormalities, chorioretinitis, or sensorineural hearing impairment until 12 months of life also [23].

As among children who develop SNHL secondary to cCMV infection, hearing loss may be present at birth or may have a delayed onset, occurring during the first years of life. Many of those babies are asymptomatic at birth. This observation underlines the need for a close follow-up of all newborns with cCMV infection, regardless of the presence of symptoms at birth, as the baby can receive serial audiological monitoring during the first years of life and up to school age to promptly detect any SNHL and proceed with rehabilitation interventions, which is able to reduce the functional deterioration resulting from deafness, significantly improving the receptive and expressive language and the socio-emotional development. In addition to all potential benefits, the use of antiviral therapy with ganciclovir and valganciclovir must consider known risks, such as neutropenia, as well as potential risks, such as gonadal toxicity and carcinogenicity, which have been observed in animal models (although no such toxicities have been demonstrated in humans at this time) [12]. Given that most cCMV-infected infants are asymptomatic, our findings on the natural history of the disease are pretty relevant; indeed, only a third of infants in our group received ganciclovir (36.9%) compared to previous data reported by Ronchi et al. (52.9%) [23]. This difference in the frequency of medical treatment in our patients has two explanations: the first is that patients with moderate to severe symptoms have been treated. The second is that our observation began in 2011, when antiviral therapy was administered almost exclusively intravenously and required the patient to be hospitalized. Therefore, it was reserved for the most serious patients. Currently, with the use of oral valganciclovir, prescribing the therapy while respecting the protocol is certainly simpler than before.

Among our patients, half of the babies without symptoms of congenital Toxoplasmosis at birth showed long-term sequelae. Although *Toxoplasma gondii* is not a newly discovered germ, there is currently no standardized treatment for congenital toxoplasmosis, and opinions on drugs and duration are sometimes conflicting. Furthermore, most drugs work against actively dividing tachyzoites and not encysted bradyzoites, making eradicating the disease more complicated. This applies to symptomatic infants but also to serologically positive but asymptomatic infants, who are often treated until the infection is excluded, usually in three to four months [24]. What is certain is that babies must be followed with frequent neurodevelopmental, ophthalmological, and auditory evaluations to evaluate the response to therapy, rule out infection when they are not symptomatic, and identify any late sequelae.

The treatment of the asymptomatic infant exposed to Syphilis in pregnancy who is unlikely to have the disease is also much discussed. These are those children whose mothers received adequate therapy before 4 weeks after delivery but for whom, in the absence of prolonged follow-up, most infectious disease specialists and even CDCs recommend benzathine penicillin G 50,000 units/kg for intramuscular (IM) dose in a single dose, even if asymptomatic [25,26]. Indeed, a late diagnosis in a newborn exposed to Syphilis changes a child that would be normal with timely therapy into a child with late and persistent intellectual disability, hearing impairment, and skeletal abnormalities. Therefore, also in this case, the need for a specific and prolonged follow-up is indisputable.

In neonates exposed to infections during pregnancy, a long-term multi-specialist follow-up is unquestionable, regardless of the type of germ and regardless of the presence of symptoms at birth. Currently, pharmacological and rehabilitative treatments are available that are effective in preventing both short- and long-term outcomes, but unfortunately, the indications for the treatment are often conflicting, especially when symptoms are not present at birth. This consideration further underlines the importance of long-term follow-up multicenter studies and the need for consensus conferences, which, based on the studies, issue shared and homogeneous recommendations to follow, especially when no signs and symptoms are present at birth.

Most moderate or severe sequelae rose in infants within the first year of life, and more than half of infected children had no sequelae in our cohort.

A new finding is the presence of a higher incidence of cognitive impairment at 24 months of follow-up in *Toxoplasma*-infected patients (14.3%). Recently, Al Malki et al. suggested that a maternal Toxoplasmosis infection could have a role in developing childhood autism linked to several points of mutation of mitochondrial and nuclear DNA [27]. Conversely, the rate of visual anomalies in congenital *Toxoplasma* infection (7.1%) was lower than those disclosed by Peyron et al. in a previous cohort of adult individuals with treated congenital toxoplasmosis (12.7%) [28].

Infants with congenital CMV infection had the highest incidence of sensorineural hearing loss (14.3%); as is well-known, among hearing-impaired children, cCMV is a common cause of sensorineural hearing loss. The mechanisms by which cCMV infection induces sensorineural deafness have been partially characterized: a pro-inflammatory reaction, the apoptosis of cochlear spiral ganglion, and vascular changes are all involved [29].

Language delay was observed in all three infections, with a higher rate in the *Toxoplasma* (35.7%) and in the cCMV group (26.1%). The association of language delay in congenital Toxoplasmosis is poorly reported in the literature, but our incidence was similar to those detected in a Brazilian cohort (26.4%) [30].

Limitations of this study include the single-center site and the small sample size. However, we compared short-term and long-term neurodevelopmental outcomes in uninfected and infected infants, always using the same objective methods: our findings could be helpful to counsel parents about these congenital infections, especially in the case of *Toxoplasma* and Syphilis, for which less data are available compared to cCMV.

## 5. Conclusions

This study confirms that infants with congenital infection who were symptomatic at birth have a worse prognosis than asymptomatic ones. Long-term sequelae may occur in infected children, also if asymptomatic at birth, up to 2–4 years of age. These results highlight the importance of the multidisciplinary follow-up that these children need, independently from the presence of overt symptoms of infection at birth and regardless of the type of congenital infection.

## Figures and Tables

**Table 1:** Definitions of congenital Cytomegalovirus, Toxoplasmosis and Syphilis infections.

	Definitions
**Cytomegalovirus**	***Symptomatic infection*** Presence of one or more of these symptoms: thrombocytopenia, petechiae, hepatomegaly, splenomegaly, intrauterine growth restriction, hepatitis (raised transaminases or bilirubin), central nervous system involvement such as microcephaly, radiographic abnormalities consistent with cytomegalovirus central nervous system disease (ventriculomegaly, intracerebral calcifications, cortical or cerebellar malformations), abnormal cerebrospinal fluid indices for age, chorioretinitis, sensorineural hearing loss, the detection of cytomegalovirus DNA in cerebrospinal fluid isolated sensorineural hearing loss (≥21 decibels), and isolation of CMV or identification of viral DNA in urine or saliva or blood. ***Asymptomatic infection*** No apparent abnormalities to suggest congenital cytomegalovirus disease, normal hearing, and isolation of CMV or identification of viral DNA in urine or saliva or blood, and detection of specific CMV IgM in blood during the first 3 weeks of postnatal life, without overt clinical symptoms of the infection.
**Toxoplasmosis**	***Symptomatic infection*** Presence of the classic triad of clinical signs: chorioretinitis, intracranial calcifications, and hydrocephalus and or microcephaly, intracranial calcifications, chorioretinitis, cataracts, convulsions, nystagmus, jaundice, petechiae, anemia, prematurity and severe intrauterine growth restriction, and the presence of positive specific IgG and/or IgM antibodies against *Toxoplasma gondii*. ***Asymptomatic infection*** Intrauterine infection without obvious signs of toxoplasmosis at birth on routine examination, with positive specific IgG and IgM antibodies against *Toxoplasma gondii*, or with an increasing specific IgG serum level.
**Syphilis**	***Confirmed Proven or Highly Probable Congenital Syphilis *** Any neonate with • an abnormal physical examination that is consistent with congenital syphilis; • a serum quantitative nontreponemal serologic titer that is fourfold (or greater) higher than the mother’s titer at delivery (e.g., maternal titer = 1:2, neonatal titer ≥ 1:8 or maternal titer = 1:8, neonatal titer ≥ 1:32); • or a positive darkfield test or PCR of placenta, cord, lesions, or body fluids or a positive silver stain of the placenta or cord. ***Possible Congenital Syphilis *** Any neonate who has a normal physical examination and a serum quantitative nontreponemal serologic titer equal to or less than fourfold of the maternal titer at delivery (e.g., maternal titer = 1:8, neonatal titer ≤ 1:16) and one of the following: • The mother was not treated, was inadequately treated, or has no documentation of having received treatment. • The mother was treated with erythromycin or a regimen other than those recommended in these guidelines (i.e., a nonpenicillin G regimen). • The mother received the recommended regimen but treatment was initiated ***Congenital Syphilis Less Likely *** Any neonate who has a normal physical examination and a serum quantitative nontreponemal serologic titer equal or less than fourfold of the maternal titer at delivery (e.g., maternal titer = 1:8, neonatal titer ≤ 1:16) and both of the following are true: • The mother was treated during pregnancy, treatment was appropriate for the infection stage, and the treatment regimen was initiated ≥30 days before delivery. • The mother has no evidence of reinfection or relapse. ***Congenital Syphilis Unlikely *** Any neonate who has a normal physical examination and a serum quantitative nontreponemal serologic titer equal to or less than fourfold of the maternal titer at delivery and both of the following are true: • The mother’s treatment was adequate before pregnancy. • The mother’s nontreponemal serologic titer remained low and stable (i.e., serofast) before and during pregnancy and at delivery (e.g., VDRL ≤ 1:2 or RPR ≤ 1:4).

**Table 2:** Clinical characteristics at birth of the 186 infants enrolled in the study.

Clinical Characteristics at Birth	Symptomatic Infected Infants (*n* = 37)	Asymptomatic Infected Infants (*n* = 82)	Uninfected Infants (*n* = 67)	*p*-Value
Gender (males), *n* (%)	23 (62.2%)	46 (56.1%)	30 (44.8%)	NS
Gestational age (week), mean ± SD	37.39 ± 3.18	38.92 ± 2.04	38.86 ± 1.50	0.001
Birth weight (g), mean ± SD	2684.51 ± 769.91	3149.04 ± 570.81	3146.95 ± 488.56	0.000
Birth weight small for gestational age, *n* (%)	8 (21.6%)	12 (14.6%)	8 (11.9%)	0.022
Head circumference (cm), mean ± SD	32.45 ± 3.02	34.37 ± 1.32	34.07 ± 1.40	0.000
Head circumference small for gestational age, *n* (%)	7 (18.9%)	6 (7.3%)	6 (9.0%)	0.028
Microcephaly, *n* (%)	5 (13.5%)	3 (3.7%)	3 (4.5%)	0.004
Cesarean section, *n* (%)	13 (35.1%)	20 (24.4%)	14 (20.9%)	NS
Apgar 5′, median (IQR)	9 (9–10)	9 (9–10)	9 (9–10)	0.019

**Table 3:** Short-term (T1: 1 year of life) outcome at follow-up in 119 infected children.

	Normal Development in 119 Infected Patients	Abnormal Development in 119 Infected Patients	
		One Sequela	Sequelae > 2
	T1 No. (%)	T1 No. (%)	T1 No. (%)
**CMV (*n* = 84)**			
*Asymptomatic at birth (n = 55)*	49/55 (89.1)	6/55 (10.9)	0/55 (0)
*Symptomatic at birth (n = 29)*	13/29 (44.8)	12/29 (41.4)	4/29 (13.8)
**TOXOPLASMA (*n* = 18)**			
*Asymptomatic at birth (n = 12)*	11/12 (91.7)	1/12 (8.3)	0/12 (0.0)
*Symptomatic at birth (n = 6)*	3/6 (50.0)	2/6 (33.3)	1/6 (16.7)
**SYPHILIS (*n* = 17)**			
*Asymptomatic at birth (n = 15)*	14/15 (93.3)	1/15 (6.7)	0/15 (0.0)
*Symptomatic at birth (n = 2)*	0/2 (0.0)	0/2 (0.0)	2/2 (100.0)
**TOTAL (*n* = 119)**			
*Asymptomatic at birth (n = 82)*	74/82 (90.2)	8/82 (9.8)	0/82 (0.0)
*Symptomatic at birth (n = 37)*	16/37 (43.2)	14/37 (37.8)	7/37 (18.9)

**Table 4:** Short-term neurodevelopmental outcomes in 119 infected infants at 1-year follow-up visit.

Type of Infection	Cognitive Delay (Score < 85)	Motor Impairment (Score < 85)	Mild SNHL	Severe SNHL	Mild Abnormal Vision	Severe Visual Impairment
CMV (*n*= 84)	3 (3.6%)	11 (13.1%)	11 (13.1%)	1 (1.2%)	2 (2.4%)	0 (0.0%)
Toxoplasma (*n* = 18)	1 (5.6%)	3 (16.6%)	1 (5.6%)	0 (0.0%)	1 (5.6%)	1 (5.6%)
Syphilis (*n*= 17)	0 (0%)	3 (17.6%)	0 (0.0%)	0 (0.0%)	0 (0.0%)	0 (0.0%)
**Total** **(*n*= 119)**	**4 (3.4%)**	**17 (14.3%)**	**12 (10.1%)**	**1 (0.8%)**	**3 (2.5%)**	**1 (0.8%)**

**Table 5:** Long-term (T2: 2–4 years of life) outcome at follow-up in 92 infected children.

	Normal Development in 92 Infected Patients	Abnormal Development in 92 Infected Patients	
		One Sequela	Sequelae > 2
	T2 No. (%)	T2 No. (%)	T2 No. (%)
**CMV (*n* = 69)**			
*Asymptomatic at birth (n = 42)*	34/42 (81.0)	8/42 (19.0)	0/42 (0.0)
*Symptomatic at birth (n = 27)*	8/27 (29.6)	14/27 (51.9)	5/27 (18.5)
**TOXOPLASMA (*n* = 14)**			
*Asymptomatic at birth (n = 8)*	4/8 (50.0)	4/8 (50.0)	0/8 (0.0)
*Symptomatic at birth (n = 6)*	3/6 (50.0)	1/6 (16.7)	2/6 (33.3)
**SYPHILIS (*n* = 9)**			
*Asymptomatic at birth (n = 7)*	5/7 (71.4)	2/7 (28.6)	0/7 (0.0)
*Symptomatic at birth (n = 2)*	0/2 (0.0)	0/2 (0.0)	2/2 (100.0)
**TOTAL (*n* = 92)**			
*Asymptomatic at birth (n = 57)*	43/57 (75.4)	14/57 (24.6)	0/57 (0.0)
*Symptomatic at birth (N = 35)*	11/35 (31.4)	15/35 (42.9)	9/35 (25.7)

**Table 6:** Long-term neurodevelopmental outcomes in 92 infected infants at 2–4 year follow-up visit.

**Type of Infection**	**Cognitive Delay (Score < 85)**	**Motor Impairment (Score < 85)**	**Mild SNHL**	**Severe SNHL**	**Mild Abnormal Vision**	**Severe Visual Impairment**	**Language Delay**
CMV (*n* = 69)	4 (5.8%)	4 (5.8%)	9 (13.0%)	1 (1.4%)	2 (2.%)	0 (0.0%)	18 (26.1%)
Toxoplasma (*n* = 14)	2 (14.3%)	2 (14.3%)	0 (0.0%)	0 (0.0%)	1 (7.1%)	1 (7.1%)	5 (35.7%)
Syphilis (*n* = 9)	1 (11.1%)	1 (11.1%)	0 (0.0%)	0 (0.0%)	0 (0.0%)	0 (0.0%)	3 (33.3%)
**Total** **(*n* = 92)**	**7 (7.6%)**	**7 (7.6%)**	**9 (9.8%)**	**1 (1.1%)**	**3 (2.3%)**	**1 (1.1%)**	**26 (28.3%)**

## Data Availability

All considered data in this study are reported in this article.
